# ASSOCIATION BETWEEN EXERCISE AND UNCONTROLLED BLOOD PRESSURE IN HYPERTENSIVE PATIENTS

**DOI:** 10.1093/geroni/igad104.2576

**Published:** 2023-12-21

**Authors:** Sinwoo Hwang, Eunhee Cho

**Affiliations:** Yonsei University College of Nursing, Seoul, Republic of Korea; Yonsei University College of Nursing, Seoul, Republic of Korea

## Abstract

Hypertension is closely associated with cerebrovascular and cardiovascular diseases, the most frequent causes of death in Korean adults. Despite its continuously improving treatment rate, it is necessary to consider patients whose blood pressure remains uncontrolled. Walking exercises are strongly recommended to lower blood pressure. This study aimed to examine the level of walking in hypertensive patients and factors that deterred walking. This cross-sectional study was conducted through secondary data analysis using data collected from the Korea National Health and Nutrition Evaluation Survey from 2016 to 2020. Participants included 1,800 hypertensive patients over the age of 19 years, whose blood pressure was not under control despite medication. Walking was defined as exercise lasting for at least 30 minutes more than five times a week. Multiple logistic regression analysis was conducted to evaluate factors that deterred walking as an exercise in hypertensive patients. Of the total sample, 63.8% patients (n=1,149) were not exercising (walking). Job and obesity were two significant factors associated with walking less. Patients who were employed or obese were more likely to not walk, compared to those unemployed (OR, 1.31; 95% CI, 1.04–1.65) or those with normal weight (OR, 1.43; 95% CI, 1.03–1.98). Patients with uncontrolled blood pressure despite medication need lifestyle modifications as well. High medication adherence should not be the reason to overlook the management of hypertensive patients. In fact, detailed management is required to identify vulnerable groups, particularly those who do not engage in even walking exercises regularly, which is a simple treatment of hypertension.

